# Neuropathy in Parkinson’s Disease Patients with Intestinal Levodopa Infusion versus Oral Drugs

**DOI:** 10.1371/journal.pone.0066639

**Published:** 2013-06-20

**Authors:** Constanze Jugel, Felicitas Ehlen, Birol Taskin, Frank Marzinzik, Thomas Müller, Fabian Klostermann

**Affiliations:** 1 Department of Neurology, Charité - University Medicine, Campus Benjamin Franklin, Berlin, Germany; 2 Department of Neurology, St. Joseph-Hospital, Berlin-Weissensee, Germany; Charité, Campus Benjamin Franklin, Germany

## Abstract

**Background:**

Severe polyneuropathy has been observed in a number of patients treated for Parkinson’s disease with Levodopa/Carbidopa intestinal gel infusion. This may reflect a rare individual complication or a systematic side effect.

**Objective:**

To investigate whether peripheral nerve function differed between patients with oral treatment versus Levodopa/Carbidopa intestinal gel infusion.

**Methods:**

In an observational design, data from median, tibial, and peroneal neurography were prospectively assessed and compared between patients with conventional drug treatment (n = 15) and with Levodopa/Carbidopa intestinal gel infusion (n = 15). The groups were matched for age and disease duration. In view of the medical risk profile for polyneuropathy, comorbidity and basic serological parameters were assessed.

**Results:**

Axonal neuropathy was common in both patient groups. However, although group differences in risk factors for polyneuropathy were not evident, neurographic abnormalities were more severe in the patients treated with Levodopa/Carbidopa intestinal gel infusion than in the orally treated patients. In the group with Levodopa/Carbidopa intestinal gel infusion, the degree of neuropathic change correlated with weight lost since therapy initiation and with the drug dose. In contrast to the axonal abnormalities, conduction velocity was found normal in both groups.

**Conclusion:**

The results are compatible with the promotion of axonal neuropathy by Levodopa/Carbidopa intestinal gel infusion. This could be due to the intrinsically high levodopa doses associated with the therapy and/or malnutritional effects from intestinal drug application. The results should be corroborated by a larger longitudinal and controlled trial.

## Introduction

Levodopa (LD) is the most common and effective drug for the suppression of motor features in Parkinson’s disease (PD). Unfortunately, long-term levodopa therapy is associated with motor complications. Thus, five to ten years after initiation of the therapy patients may experience debilitating fluctuations between hypo- and hyperkinetic states, with increasingly short periods of acceptable conditions in between [1]. If alternative supportive drug approaches fail to alleviate this problem, the route of levodopa administration can be changed to a direct intestinal application to bypass irregular gastric emptying. This so-called Levodopa/Carbidopa Gel Infusion (LCIG/Duodopa®) is carried via a jejunal tube through a percutaneous endoscopic gastrostomy by a wearable pump system [2].

LCIG treatment effectively reduces motor fluctuations, and typically allows to simplify the drug regimen from poly to monotherapy [3]. Duodopa® has now become available in most European and some non-European countries as an orphan drug for PD patients with severe motor fluctuations. Common side effects of the therapy, such as tube dislocation, are of a technical nature and can therefore usually be handled rather easily [4]. However, in addition to system-related complications, severe cases of polyneuropathy have been reported under LCIG treatment. To date, it remains controversial whether these incidents reflect generic neuropathogenic effects of LCIG (or, less specifically, of LD), a rare individual side effect, or coincidence. One reason for this uncertainty is that the size of patient populations in which the reported cases occurred is largely unknown. Another reason is that the course of polyneuropathies under LCIG was partly reminiscent of putatively treatment-unrelated autoimmune diseases, such as chronic inflammatory demyelinating neuropathy (CIDP) or Guillain-Barré syndrome (GBS), though mostly being axonal in nature [5]–[9].

Against this background, we neurographically assessed sensorimotor parameters of peripheral nerve function in patients treated with LCIG at our PD outpatient clinic. The results were compared to the respective values in orally treated PD patients matched for age and disease duration. Common risk factors for neuropathy were clinically and serologically ascertained in both groups. We posited that group differences indicative for a common type of polyneuropathy, i.e. axonal, distally distributed and predominantly sensory, were in line with general neuropathogenic effects of Duodopa®. On the other hand, we expected that if polyneuropathy was a rare, individual, all-or-nothing side effect, or was even unrelated to the treatment, the neurophysiological assessment of peripheral nerve function should hardly differ between groups.

## Patients and Methods

30 PD patients from the outpatient clinic of the Charité, Campus Benjamin Franklin, participated in this observational study. They were either treated with oral drugs (n = 15, m/f = 9/5, 69±8 years) or LCIG (n = 15 m/f = 11/4, 69±8 years). In both groups, clinical and neurophysiological data were prospectively assessed after an average disease duration of 14 years, and in the LCIG group after 736±420 days on pump treatment (range: 204 to 1447 days). Per patient all assessments were done on the same day. The LCIG patients were recruited out of 25 available candidates at our site and defined the selection of the orally treated patients who should match for age, duration of PD and the motor UPDRS under the current medication. The main disease-related group difference of the participants was the higher severity of motor fluctuations in LCIG patients before pump treatment with off states during at least thirty percent of waking time. In the orally treated group, only few patients, reluctant to therapy escalation by DBS or pump treatment, fulfilled this criterion. All patients gave written informed consent to participate in the trial, approved by the local Ethics Committee of the Charité, section IV at the Campus Benjamin Franklin (study sign EA4/021/12).

The patients underwent a physical exam to determine the motor score of UPDRS (part III), the Hoehn-&-Yahr stage and polyneuropathic complaints according to the modified Toronto Clinical Neuropathy Score (ranging from 0 to 33 points per body side indicating no and, respectively, maximal neuropathic impairment) [10]. General medical risk factors for polyneuropathy (i. e. diabetes, alcoholism, rheumatic diseases, thyroid disease, specific infections, malignant diseases, malnutritional conditions, exposition to neurotoxic substances or drugs) were assessed by interview. Patients were asked about weight loss, which is supposed to be a specific LCIG side effect (and is possibly a risk factor for neuropathy), since initiation of LCIG, and since 2006 in the orally treated group (as the earliest possible year of LCIG treatment). Finally, blood samples were taken to assess the basic neuropathogenic risk profile (HbA1c, one-hr/two-hr ESR, creatinine, AST, ALT, GGT, TSH, protein, albumin, serum electrophoresis, antinuclear antibodies). To explore differences in the demographic, clinical and laboratory data of LCIG versus orally treated patients two-sided Wilcoxon-tests (p-level <0.05) were used, apart from the analysis of the gender distribution between the groups performed by the Mann-Whitney-U-Test.

Neurographically, compound motor action potentials (cMAP) after bilateral median, tibial, and peroneal nerve stimulation were recorded respectively from the Musculi abductor pollicis, flexor hallucis brevis, and extensor digitorum brevis. Nerve conduction velocity was calculated from the latency difference for the potentials elicited from proximal (median nerve: cubital fossa/tibial, peroneal nerve: respectively mid and, lateral popliteal fossa) versus distal stimulation sites (median nerve: wrist; tibial, peroneal nerve: respective ankle points; innervation anomalies of median and peroneal nerves were excluded), referenced to the distance between the stimulation points per nerve. For sensory neurography, compound nerve action potentials (cNAP) were recorded from the end plantar index finger after median nerve stimulation at the wrist and from an area dorsal to the lateral malleolus after sural nerve stimulation about fourteen centimetres proximal to this site. All recordings were performed bilaterally with stick electrodes (3.5 cm cathode-to-anode distance) and a Medtronic Keypoint® system. To minimise the influence of the surrounding temperature on nerve conduction, the neurographic tests were principally performed as the last examination, after the patients had already been at a room temperature of 20 degrees Celcius at least one hour. To minimise a possible bias in the data, the person, who performed the neurophysiological diagnostics at our site and who was not involved in any aspect of the treatment, carried out the meaurements and evaluations. According to the period of patient recruitment, data were collected over one year upon request of routine neurophysiological diagnostics for potential polyneuropathy.

Statistically, we aimed to analyse the overall nerve state in view of group differences, and to assess potential cofactors. Since the measured amplitudes and conductions velocities met criteria for parametric testing due to Kolmogorov-Smirnow and Levene testing, they were subjected to three-factorial ANOVAs (between subject factor ‘Group’ [2 levels: oral treatment/LCIG], within subject factors ‘Nerve’ [5 levels: motor median/tibial/peroneal/sensory median/sural] and ‘Side’ [2 levels: left/right]). In the event of sphericity violations, Greenhouse-Geisser corrections were performed. Post-hoc comparisons were performed with two-sided t-tests.

In an attempt to identify potential risk factors for neuropathy, Spearman rank correlations with various parameters were calculated for the neurographic results. Therefore, the number of abnormally low potentials in each patient was ascertained (minimum = 0, no cMAP/cNAP abnormal/maximum = 10, all five cMAPs/cNAPs low on either side) based on the lab-specific, age-adjusted cut-off values for the measured potentials (4 mV for median, 3 mV for tibial and peroneal motor, 12 µV for median sensory and 8 µV for sural potentials). This value was used as the dependent variable in correlations to (i) age, (ii) disease duration, (iii) disease severity (as reflected by the motor UPDRS), (iv) weight loss, and (v) daily as well as cumulative LD dose (taken during the PD treatment, estimated from the medical records)/LD equivalence dose (LED, the calculated efficacy of PD medication, expressed in milligram LD) [11]. For comparing the correlations in the LCIG versus orally treated group, Fisher-Z-transformations of the calculated values [Z_Fisher_ = 1/2 ln((1+r)/(1-r))] were performed and the p-value, reflecting the probability (σ) of erroneously assuming group differences, was calculated [σ_(ZFisher1-ZFisher2)_ = √(1/(n_1_-3)+1/(n_2_-3))] [12].

## Results

Patients treated with oral drugs and LCIG did not differ with respect to age, duration of disease, the Hoehn-&-Yahr stage and the motor UPDRS on medication. Orally treated patients always had a co-medication of LD and other PD drugs (typically entacapone, amantadine, or one of various dopamine agonists), whereas the dopaminergic medication only comprised LD in the LCIG group (with the exception of one patient who additionally took entacapone for COMT inhibition). The LED was on average twenty-two percent lower in the orally treated group than in the LCIG treated group. While this difference was not yet statistically significant, the LD dose was, as expected, significantly higher in the LCIG than in the orally treated group (cf. Table 1).

**Table 1:** Patient characteristics in LCIG and orally treated patients.

	LCIG (n = 15)	orally treated (n = 15)	*p*-value
Gender [m/f]	11/4	9/6	0.53
Age [years]	69±8	69±8	0.95
BMI [kg/m2]	22.8±3.6	24.3±5.0	0.38
PD duration	14±3	14±4	0.50
Hoehn & Yahr	3.5±0.7	3.3±0.6	0.4
UPDRS, part III	30.1±5.4	30.2±8.7	0.96
Levodopa dose [mg]	1909±540	1047±514	0.001
LED [mg]	1961±640	1526±520	0.06
Time on LCIG [days]	736±420	n.a.	

BMI: Body Mass Index; LCIG: Levodopa Carbidopa Intestinal Gel; LED: levodopa equivalence dose; PD: Parkinson’s disease; UPDRS: Unified Parkinson’s Disease Rating Scale.

* p≤0,05;

Data provided as mean values ± standard deviation (where applicable).

Groups did not differ significantly regarding the modified Toronto Clinical Neuropathy Score, although on average the impairment appeared somewhat higher in LCIG patients (8.4±6.2/9.1±7.2 for the left/right side) than in the orally treated patients (5.7±4.3/5.5±3.6 for the left/right side). In terms of medical risk factors for polyneuropathy, in the orally treated group one patient had a monoclonal gammopathy (Morbus Waldenström), one a metastasis from a prostate carcinoma (treated with cyproteronacetate), one a well-controlled hypothyroidism (treated with 50 µg levothyroxine), and one suffered of chronic compensated renal failure. In the LCIG group, one patient had type II diabetes (orally treated with glimeperide, normal HbA1c), and one a well-controlled hypothyroidism (also treated with 50 µg levothyroxine per day). The results from the blood tests were not significantly different between groups. The number of values below or above cut-off was similar between groups, i.e. 27 and 23 in LCIG and orally treated patients respectively, out of 240 values per group (due to the assessment of 16 parameters in 15 patients). Group-specific clusters of abnormal values in particular parameters were not discernible, apart from a certain deviation towards low protein values in the LCIG versus orally treated group (5 patients versus 1 patient). An overview of the laboratory results is provided in Table 2.

**Table 2:** Laboratory results in LCIG and orally treated patients.

	LCIG (n = 15)	orally treated (n = 15)	*p*-value
ESR 1 h	15.3±10.7	21.8±23.3	0.38
2 h	29.9±16.8	33.8±25.6	0.65
HbA_1_c [%]	5.8±0.3	5.7±0.7	0.52
TSH [mU/l]	1.1±0.7	1.7±1.3	0.12
Creatinine [mg/dl]	0.8±0.1	1.0±0.4	0.11
ALT [U/l]	13.5±14.9	14.9±9.3	0.76
AST [U/l]	25.9±11.4	21.8±5.7	0.23
GGT [U/l]	17.7±7.9	31.2±21.6	0.06
Protein [g/dl]	6.6±0.9	7.1±0.6	0.1
Albumin [g/dl]	4.4±0.7	4.4±0.2	0.53
Albumin [%]	58.9±4.5	58.1±4.7	0.69
Alpha-1 globuline [%]	4.5±0.7	4.3±0.8	0.38
Alpha-2 globuline [%]	10.4±1.1	10.8±2.1	0.53
Beta globuline [%]	11.1±1.1	10.9±1.4	0.66
Gamma globuline [%]	15.2±3.6	15.9±6.6	0.76
ANA titre	1∶53.3±1∶167.4	1∶160.0±1∶255.3	0.6

ESR: erythrocyte sedimentation rate; HbA_1_c: glycated haemoglobin, TSH: thyroid-stimulating hormone; ALT: alanine transaminase, AST: aspartate transaminase; GGT: gamma-glutamyltransferase; ANA: anti-nuclear antibodies last column: p-values for group comparisons per parameter Data provided as mean values ± standard deviation.

Coming from an almost identical Body Mass Index (BMI; orally treated/LCIG group: 24.9±5.2/25.2±3.2), the reported weight loss since the start of the LCIG therapy was 7.2±5.7 kilograms, and 2±10 kilograms since 2006 in the orally treated patients, but this difference was not significant (the current BMI values and the changes from the reported initial BMI were not significant between the groups; BMI in orally treated/LCIG group: 24.3±5/22.8±3.6).

With respect to clinical symptoms of polyneuropathy, the majority of the patients (10 orally treated/11 treated with LCIG) had mild to moderate distally distributed hypesthesia, particularly evident as decreased tibial, ankle and foot vibrotactile sense. Neuropathic pain was a relatively common complaint, more often categorised as severe in the LCIG than in the orally treated patients (mild: 7 orally treated patients, 6 LCIG patients; interfering with activities of daily living: 1 orally treated patient, 5 LCIG patients). Five patients per group had additional motor symptoms, apparent as slightly reduced foot force. For most patients this was not relevant for gait functions, apart from one patient in the LCIG group with a bilateral moderate drop foot.

According to neurophysiological criteria, ten out of fifteen patients had sensory and five additional motor abnormalities in the orally treated group, concerning at least one nerve potential. In the LCIG group, all patients had at least one abnormal sensory potential and ten at least one abnormal motor potential.

The ANOVA of the neurophysiological results revealed that ‘Group’ was a main factor for amplitudes [*F*(1, 28) = 9.1, p<.01], i.e. LCIG and orally treated patients differed with respect to the magnitude of the measured motor and sensory nerve potentials. Further, an interaction of this main effect with ‘Nerve’ was shown [Group x Nerve: *F*(4, 112) = 3.6, p<.05], meaning that neurographic differences were not equally distributed. To delineate which nerves mainly contributed to the overall group difference, post-hoc comparisons were conducted. Bilateral sensory potentials from the median (left/right: p = 0.023/0.039; across sides: p = 0.02) and sural (left, right: p = 0.002/0.01; across sides p = 0.003) nerves as well as the peroneal motor potentials (left, right: p = 0.006/0.04; across sides: p = 0.006) were significantly lower in LCIG patients than in orally treated PD patients. No significant group differences were obtained for median and tibial motor potentials. As expected, ‘Side’ was not a (co-)factor for the neurographic results.

No significant group differences were obtained with respect to nerve conduction velocities which were normal in LCIG and orally treated patients (expected ≥ fifty and forty m/s for upper and lower extremity nerves respectively). An overview of the values is provided in Table 3.

**Table 3:** Sensory and motor potentials from neurography in LCIG and orally treated patients.

	LCIG Group	orally treated Group	*p-*value
Median motor [mV]	6.5±2.8	7.3±2.4	0.52
Median motor [m/s]	55.4±4.3	53.6±5.5	0.24
Median sensory [µV]	10.5±9.4	18.6±9.5	0.02
Median sensory [m/s]	52.7±6.6	51.7±7.0	0.69
Peroneal motor [mV]	2.2±1.8	4.2±1.8	0.006
Peroneal motor [m/s]	44.2±3.4	45.8±4.9	0.31
Tibial motor [mV]	3.5±3.4	4.5±3.1	0.41
Tibial motor [m/s]	45.1±4.2	44.7±3.9	0.72
Sural sensory [µV]	2.3±2.7	6.7±4.3	0.003
Sural sensory [m/s]	52.9±5.8	50.9±5.7	0.54

data provided as mean values from bilateral measurements ± standard deviation p-values for t-tests over both sides.

**Figure 1:**
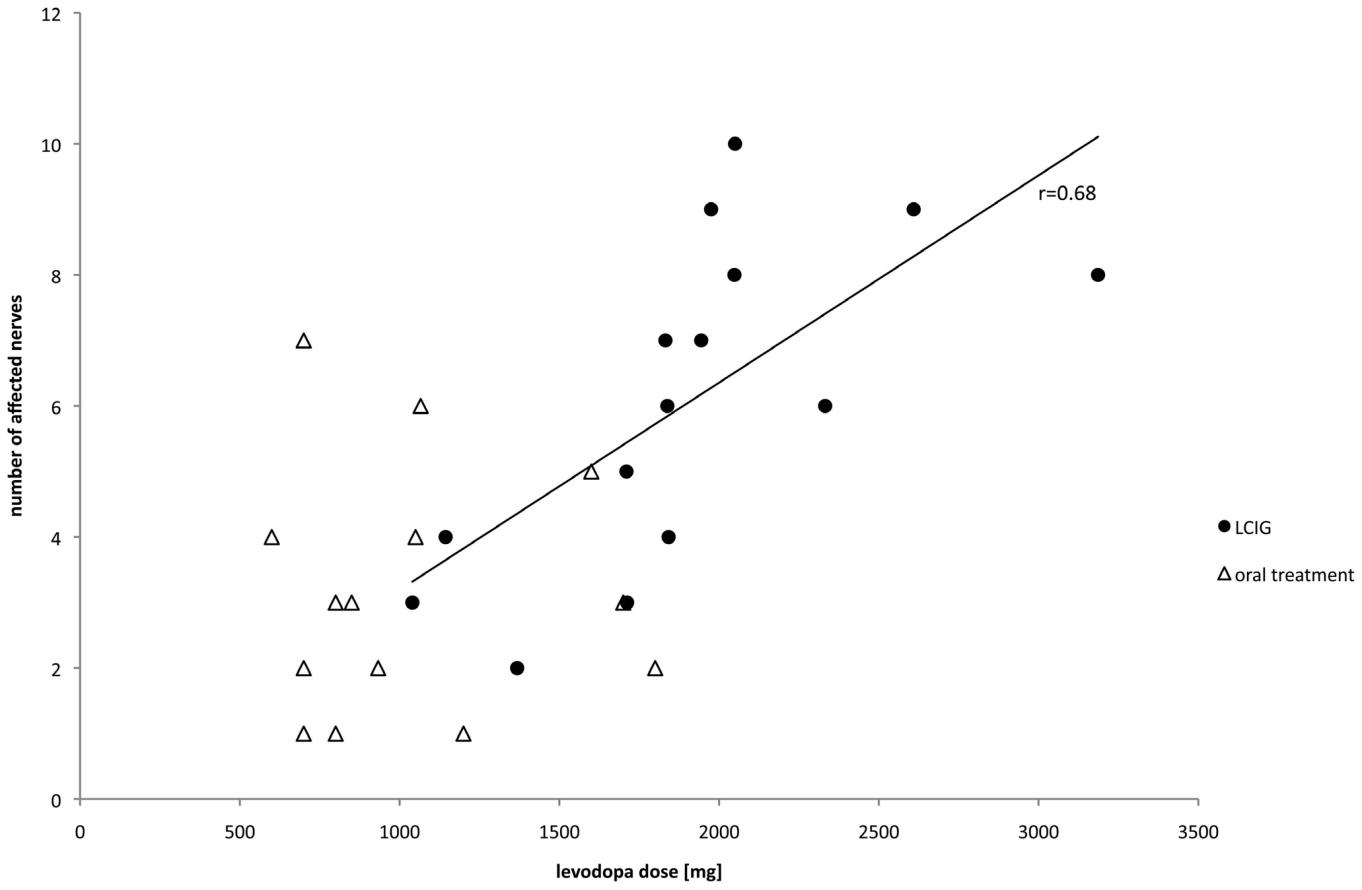
Correlation of daily levodopa dose and the number of neurographically impaired nerves. The daily levodopa dose is indicated on the abscissa, the number of neurographically impaired nerves on the ordinate. Triangles and dots reflect the values of the orally treated and LCIG patients respectively. The regression line is provided for the data from the LCIG group for which the correlation coefficient (r) was significantly higher than in the orally treated group.

The number of neurographically impaired nerves was 6.1±2.5 in the LCIG patients and 3.1±1.7 in the orally treated patients. Significant correlations between this parameter with the current as well as the cumulative LD and LED were found in the LCIG group, but not in the orally treated group (exemplified in Figure 1). Furthermore, correlations with weight loss (since initiation of the pump therapy) were only identified in the LCIG group. The calculated correlations for the daily and cumulative LD as well as for the cumulative LED differed significantly between the groups. These results are summarised in Table 4.

**Table 4:** Correlations between the number of impaired nerves and patient parameters.

	LCIG	oral treatment	Δ (*p-*value)
LD, daily	0.68*	−0.03	0.018
LD, cumulative	0.62*	−0.15	0.016
LED, daily	0.68*	0.33	0.117
LED, cumulative	0.66*	0.11	0.047
weight loss	0.60*	0.16	0.096
age	0.39	0.22	0.322
disease duration	0.32	−0.04	0.181
disease severity	0.13	0.04	0.412

The first two data columns provide the correlation values per parameter in each group. The last column indicates the p-values for the comparisons of the correlation coefficients between the groups (reflecting the probability of erroneously assuming distinct correlations in LCIG versus orally treated patients).

LD: levodopa dose.

LED: levodopa equivalence dose.

numbers: Spearman rank correlation (with asterisk: p<0.05).

Δ (p-value): probability of erroneously assuming different correlations between the groups.

## Discussion

LCIG treated PD patients had significantly lower compound action potentials than patients receiving oral medication, particularly regarding the peroneal, sensory median, and sural nerves. Conduction velocities were normal in both groups. The patients’ age and PD duration were almost identical. General group differences between the medical risk profiles for polyneuropathy were not evident.

Although there was no significant group difference in the modified Toronto Clinical Neuropathy Score, the results are of interest with respect to putative neuropathogenic risks of LD and, in particular, LCIG [8]. So far, reports on polyneuropathy under LCIG have been casuistic, mostly describing severe conditions reminiscent of CIDP or GBS [3], [5]. Whether these cases reflect a rare individual side effect based on some unknown predisposition, or extreme phenotypes in a continuous spectrum of neuropathogenic LD or LCIG actions, is essentially unknown, but the current data appear to favour the latter possibility.

It has been shown that polyneuropathy is a common feature in long-term PD patients [13]–[17]. In line with this, most patients studied had various abnormal cNAP results, including the orally treated group. Besides disease-related neurodegenerative processes, metabolic LD effects have been assumed to be neuropathogenic, e. g., the accumulation of methyl-malonic acid or homocysteine and the increased demand of nerve nutrients (B vitamins and folate) in LD degradation. However, consistent correlations between current and cumulative LD doses and neuropathy indices were found only in the LCIG group (regardless of a possibly slightly advanced disease state in LCIG patients whose LED was some twenty percent higher than in the orally treated group).

A couple of factors might contribute to this result. First, the LD dose was significantly lower in the patients with oral medication than with LCIG and, accordingly, nonlinear dosing effects above critical threshold values might only have prevailed in the Duodopa® group (which would be a particular problem of LCIG as LD monotherapy). Further, the present versus absent associations of PD drug doses with nerve damage in patients with LCIG and oral treatment respectively could indicate that LD is not or not solely relevant for an acceleration of polyneuropathy under Duodopa®. In this regard, any effect related to the application rate of the drug might underlie the correlations identified. For example, intestinal accumulation of the viscous gel could hamper jejunal membrane functions which could promote previously described B vitamin and folate deficiencies and run counter to the increased demand of these nutrients in LD treatment. Worthwhile to note similar cases of neuropathy have been observed after surgical resection of the upper jejunum and bariatric operations [18]–[20]. Such a mechanism would also be in line with the correlation of the reported weight loss and neuropathic damage obtained in LCIG patients.

The present study was motivated by recent reports on neuropathy under Duodopa®. Since no systematic data on this relevant issue are available so far, we performed a first one-step comparison between orally treated and LCIG patients. The thus assessed preliminary data can be seen as an incentive for a larger and controlled prospective trial in which the development of neuropathies under either treatment should be precisely monitored and a number of limitations of the current study trial could be addressed. For example, we cannot completely rule out whether the degree of neuropathy varied between the study groups independently of the treatment form, since we do not have neurographic baseline data of the LCIG patients prior to pump treatment. Thus, the correlational differences between the groups are suggestive of LCIG specific neuropathogenic effects, but do not prove them. Further, uncertainties from methodological constraints, such as the lack of glucose tolerance testing in the screening for neuropathic risk factors or of skin temperature control for nerve conduction studies, could be sorted out. Particular attention will have to be paid to the development of vitamin deficiencies under LCIG as a candidate factor of neuropathy. This was not assessed in the given context because the patients studied were ‘pragmatically substituted’ by cobalamin and folate at different dates after LCIG initiation, although respective blood levels had not been determined. Thus, vitamin supplementation which is now often done right from the beginning of LCIG treatment [8] should be put to the test by prospectively sampling these data together with clinical and neurographic parameters in order to delineate the correct indication, effects, and timing of this procedure.

For the time being, we propose that the handling of LCIG as an important tool in the treatment of advanced PD should comprise screening and follow-up exams for polyneuropathy, regular determination of body weight, B vitamin and folate levels and, where appropriate, the supplementation of these nutrients.

## References

[1] Oertel WH, Wolters E, Sampaio C, Gimenez-Roldan S, Bergamasco B, et al (2006) Pergolide versus levodopa monotherapy in early Parkinson’s disease patients: The PELMOPET study. Mov Disord 21: 343–353. 16211594 10.1002/mds.20724

[2] Nyholm D, Nilsson Remahl AI, Dizdar N, Constantinescu R, Holmberg B, et al (2005) Duodenal levodopa infusion monotherapy vs oral polypharmacy in advanced Parkinson disease. Neurology 64: 216–223. 15668416 10.1212/01.WNL.0000149637.70961.4C

[3] Antonini A, Isaias IU, Canesi M, Zibetti M, Mancini F, et al (2007) Duodenal levodopa infusion for advanced Parkinson’s disease: 12-month treatment outcome. Mov Disord 22: 1145–1149. 17661426 10.1002/mds.21500

[4] Devos D, French DSG (2009) Patient profile, indications, efficacy and safety of duodenal levodopa infusion in advanced Parkinson’s disease. Mov Disord 24: 993–1000. 19253412 10.1002/mds.22450

[5] Klostermann F, Jugel C, Muller T, Marzinzik F (2012) Malnutritional neuropathy under intestinal levodopa infusion. J Neural Transm 119: 369–372. 21786115 10.1007/s00702-011-0689-3

[6] Manca D, Cossu G, Murgia D, Molari A, Ferrigno P, et al (2009) Reversible encephalopathy and axonal neuropathy in Parkinson’s disease during duodopa therapy. Mov Disord 24: 2293–2294. 19795477 10.1002/mds.22807

[7] Meppelink AM, Nyman R, van Laar T, Drent M, Prins T, et al (2011) Transcutaneous port for continuous duodenal levodopa/carbidopa administration in Parkinson’s disease. Mov Disord 26: 331–334. 20960486 10.1002/mds.23408

[8] Santos-Garcia D, de la Fuente-Fernandez R, Valldeoriola F, Palasi A, Carrillo F, et al (2012) Polyneuropathy while on duodenal levodopa infusion in Parkinson’s disease patients: we must be alert. Journal of Neurology 259: 1668–1672. 22270132 10.1007/s00415-011-6396-z

[9] Urban PP, Wellach I, Faiss S, Layer P, Rosenkranz T, et al (2010) Subacute Axonal Neuropathy in Parkinson’s Disease with Cobalamin and Vitamin B6 Deficiency Under Duodopa Therapy. Movement Disorders 25: 1748–1752. 20740570 10.1002/mds.23342

[10] Bril V, Tomioka S, Buchanan RA, Perkins BA, Grp mS (2009) Reliability and validity of the modified Toronto Clinical Neuropathy Score in diabetic sensorimotor polyneuropathy. Diabetic Medicine 26: 240–246. 19317818 10.1111/j.1464-5491.2009.02667.x PMC2871179

[11] Tomlinson CL, Stowe R, Patel S, Rick C, Gray R, et al (2010) Systematic review of levodopa dose equivalency reporting in Parkinson’s disease. Mov Disord 25: 2649–2653. 21069833 10.1002/mds.23429

[12] Bortz J (1993) Statistik für Sozialwissenschaftler. Berlin: Springer.

[13] Tison F, Le Masson G (2011) Parkinson disease, L-dopa, and neuropathy Did we miss something? Neurology 77: 1938–1939. 22049202 10.1212/WNL.0b013e31823de107

[14] Muller T, Renger K, Kuhn W (2004) Levodopa-associated increase of homocysteine levels and sural axonal neurodegeneration. Archives of Neurology 61: 657–660. 15148140 10.1001/archneur.61.5.657

[15] Rajabally YA, Martey J (2011) Neuropathy in Parkinson disease Prevalence and determinants. Neurology 77: 1947–1950. 22049200 10.1212/WNL.0b013e31823a0ee4

[16] Toth C, Breithaupt K, Ge S, Duan Y, Terris JM, et al (2010) Levodopa, Methylmalonic Acid, and Neuropathy in Idiopathic Parkinson Disease. Annals of Neurology 68: 28–36. 20582991 10.1002/ana.22021

[17] Toth C, Brown MS, Furtado S, Suchowersky O, Zochodne D (2008) Neuropathy as a Potential Complication of Levodopa Use in Parkinson’s Disease. Movement Disorders 23: 1850–1859. 18785232 10.1002/mds.22137

[18] Aasheim ET, Hofso D, Hjelmesaeth J, Sandbu R (2008) Peripheral Neuropathy and Severe Malnutrition following Duodenal Switch. Obesity Surgery 18: 1640–1643. 18463930 10.1007/s11695-008-9539-2

[19] Juhasz-Pocsine K, Rudnicki SA, Archer RL, Harik SI (2007) Neurologic complications of gastric bypass surgery for morbid obesity. Neurology 68: 1843–1850. 17515548 10.1212/01.wnl.0000262768.40174.33

[20] Thaisetthawatkul P, Collazo-Clavell ML, Sarr MG, Norell JE, Dyck PJB (2004) A controlled study of peripheral neuropathy after bariatric surgery. Neurology 63: 1462–1470. 15505166 10.1212/01.wnl.0000142038.43946.06

